# The changes of neuroactivity of Tui Na (Chinese massage) at Hegu acupoint on sensorimotor cortex in stroke patients with upper limb motor dysfunction: a fNIRS study

**DOI:** 10.1186/s12906-023-04143-0

**Published:** 2023-09-21

**Authors:** Yu-Feng Chen, Meng-Chai Mao, Guang-Yue Zhu, Cheng-Cheng Sun, Jing-Wang Zhao, Hao-Xiang He, Yu-Hui Chen, Dong-Sheng Xu

**Affiliations:** 1 Department of Massage, Hangzhou Hospital of Traditional Chinese Medicine Affiliated to Zhejiang Chinese Medical University, Hangzhou, Zhejiang China; 2 School of Rehabilitation Science, Shanghai University of Traditional Chinese Medicine, Shanghai, China; 3 Engineering Research Center of Traditional Chinese Medicine Intelligent Rehabilitation, Ministry of Education, Shanghai, China; 4 The Second Rehabilitation Hospital of Shanghai, Shanghai, China; 5 Rehabilitation Medical Center, Tongji Hospital Affiliated to Tongji University School of Medicine, Shanghai, China; 6 Department of Intensive Rehabilitation, Shanghai Third Rehabilitation Hospital, Shanghai, China; 7 Department of Internal Neurology, Tongji Hospital, Tongji University, Shanghai, China; 8 Department of Rehabilitation, Shuguang Hospital, Shanghai University of Traditional Chinese Medicine, Shanghai, China

**Keywords:** Tui Na (Chinese massage), Functional near-infrared spectroscopy (fNIRS), Stroke, Sensorimotor neural circuits, Neuroactivity

## Abstract

**Background:**

Tui Na (Chinese massage) is a relatively simple, inexpensive, and non-invasive intervention, and has been used to treat stroke patients for many years in China. Tui Na acts on specific parts of the body which are called meridians and acupoints to achieve the role of treating diseases. Yet the underlying neural mechanism associated with Tui Na is not clear due to the lack of detection methods.

**Objective:**

Functional near-infrared spectroscopy (fNIRS) was used to explore the changes of sensorimotor cortical neural activity in patients with upper limb motor dysfunction of stroke and healthy control groups during Tui Na Hegu Point.

**Methods:**

Ten patients with unilateral upper limb motor dysfunction after stroke and eight healthy subjects received Tui Na. fNIRS was used to record the hemodynamic data in the sensorimotor cortex and the changes in blood flow were calculated based on oxygenated hemoglobin (Oxy-Hb), the task session involved repetitive Tui Na on Hegu acupoint, using a block design [six cycles: rest (20 seconds); Tui Na (20 seconds); rest (30 seconds)]. The changes in neural activity in sensorimotor cortex could be inferred according to the principle of neurovascular coupling, and the number of activated channels in the bilateral hemisphere was used to calculate the lateralization index.

**Result:**

1. For hemodynamic response induced by Hegu acupoint Tui Na, a dominant increase in the contralesional primary sensorimotor cortex during Hegu point Tui Na of the less affected arm in stroke patients was observed, as well as that in healthy controls, while this contralateral pattern was absent during Hegu point Tui Na of the affected arm in stroke patients. 2. Concerning the lateralization index in stroke patients, a significant difference was observed between lateralization index values for the affected arm and the less affected arm (*P <* 0.05). Wilcoxon tests showed a significant difference between lateralization index values for the affected arm in stroke patients and lateralization index values for the dominant upper limb in healthy controls (*P <* 0.05), and no significant difference between lateralization index values for the less affected arm in stroke patients and that in healthy controls (*P =* 0.36).

**Conclusion:**

The combination of Tui Na and fNIRS has the potential to reflect the functional status of sensorimotor neural circuits. The changes of neuroactivity in the sensorimotor cortex when Tui Na Hegu acupoint indicate that there is a certain correlation between acupoints in traditional Chinese medicine and neural circuits.

## Background

Stroke is a serious threat to the safety and quality of life in the world, and the rate of disability after stroke is relatively high. Most patients have motor dysfunction, especially upper limb (UL) motor dysfunction [1]. UL movement is delicate and complex, and its unsatisfactory recovery can reduce patients’ activities of daily life and affect the living quality of patients and their families [2]. Therefore, UL motor dysfunction is a key and difficult point in the process of rehabilitation after stroke.

Tui Na (Chinese massage) is a therapy that uses different manipulations to act on specific parts of the body to achieve the purpose of prevention and treatment of diseases. Skilled and appropriate manipulations can positively affect the nervous, circulatory, motor, and other systems of the body [3]. Tui Na is well received because of its simple operation, safety, and wide range of applications. However, the essence and structural basis of the meridian guiding Tui Na practice have not been scientifically clarified, which has become the "bottleneck" of Tui Na’s inheritance and innovation [4]. Researchers have found that there may be a relationship between neural circuits and the meridian system [5, 6]. The sensorimotor neural circuits are an important way All the stroke subjects were the first unilateral ischemic stroke patients for the human body to perceive external stimuli and result in a series of physiological and pathological changes. The normal function of the structure of the central nervous system depends to a large extent on the normal transmission of sensory information. The human body receives different stimuli through various receptors distributed in the skin, muscles, and joints to produce various types of sensation [7, 8].

Studies have shown that Tui Na can improve post-stroke motor dysfunction through sensory ascending stimulation [9, 10], but the related mechanism is unclear due to the lack of detection methods. fNIRS [11] (functional near-infrared spectroscopy) is an emerging neuroimaging technique, which indirectly reflects the intensity of neural activity by detecting the changes in blood oxygen activity in the cerebral cortex. fNIRS has a good temporal resolution (sampling rate up to dozens of Hz) and spatial positioning ability (centimeter scale), as well as portability, economy, and good ecological validity [12]. In recent years, it has been widely used in neuroscience [13, 14], clinical research [15, 16], brain-computer interface [17, 18] and other fields and made some progress.

In this research, fNIRS was used to reflect the changes of neuroactivity in the sensorimotor cortex during Tui Na Hegu (LI4, large intestine 4) acupoint in patients with UL motor dysfunction after stroke, and to explore the correlation between acupoints in traditional Chinese medicine and neural circuits. The Hegu point is commonly used in Tui Na therapy, located between the hand’s first and second metacarpal bones, with a relatively large corresponding position in the cerebral cortex, and was easily observed by fNIRS. Therefore, the Hegu point was chosen as a representative point for the study.

## Materials and methods

### Participants

A total of 18 subjects were recruited, including 10 patients with ischemic stroke and 8 healthy controls without neurological or mental illness. All the stroke patients were unilateral, ischemic, and first-onset, and all were right-handed. The clinical information of stroke patients is shown in Table 1. Before participating in the experiment, all subjects fully understood the purpose of the study and signed an informed consent form. This study was approved by the Institutional Ethics Committee of Shanghai Third Rehabilitation Hospital (approval No. SH3RH-2021-EC-012) on December,16th, 2021.

**Table 1:** Clinical information of stroke patients

Patients	Sex	Age	Time of stroke	Hemiparesis side	FMA-UE
1	F	66	6 months	L	22
2	M	43	2 months	L	26
3	M	42	3 months	R	25
4	M	60	3 months	L	30
5	M	56	5 months	R	32
6	M	54	4 months	R	28
7	M	69	3 months	R	25
8	F	54	5 months	L	29
9	M	63	5 months	R	35
10	M	73	3 months	R	36

*M* Male, *F* Female, *L* Left, *R* Right, *FMA-UE* UL motor function evaluation

### Equipment and method

**Fig. 1:**
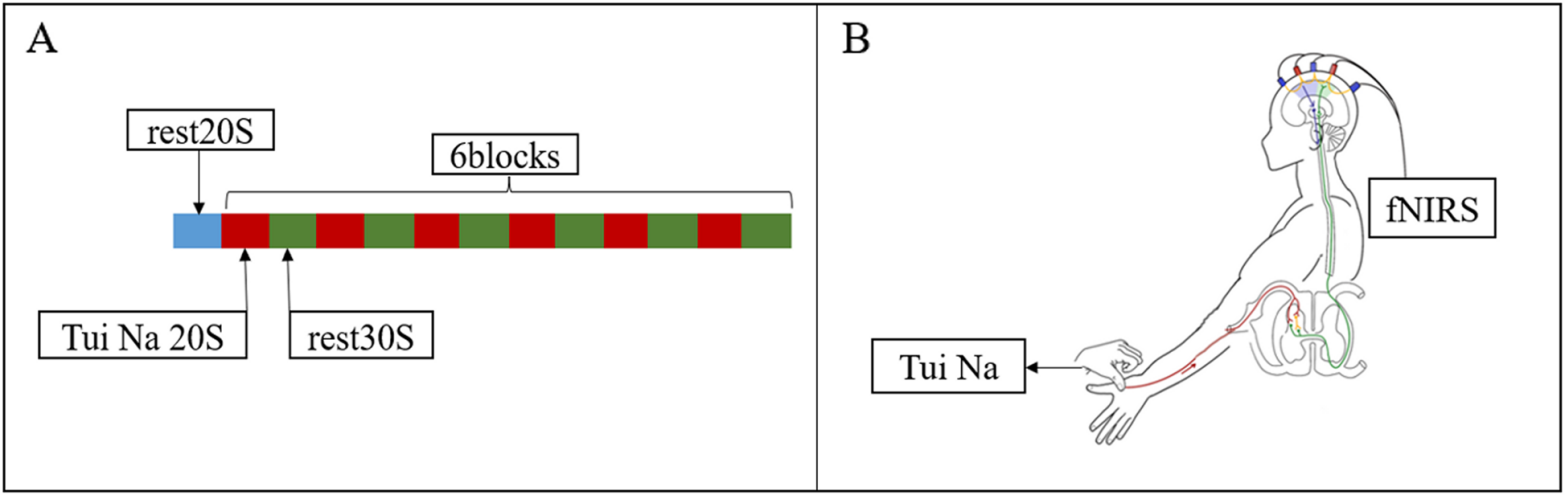
fNIRS block design (**A**) and Tui Na paradigm (**B**)

During the experiment, the subjects were asked to lie on the bed and avoid unnecessary physical activities, such as blinking, frowning, and speaking. Participants were required to wear fNIRS headcap which covered the bilateral frontal and parietal regions. The headcap was adjusted to mount the optodes tightly onto the head of participants to make sure the high quality of fNIRS signal acquisition. fNIRS recording started when the signal was stable. The task session involved repetitive Tui Na on Hegu acupoint, using a block design [six cycles: rest (20 seconds); Tui Na (20 seconds); rest (30 seconds)] (Fig. 1a). The one-finger Zen manipulation was used and operating essentials are as follows: the performer exerts the tip of the thumb on the Hegu acupoint of the subject’s hand, the thumb is straight, the metacarpophalangeal joint and interphalangeal joint of the remaining four fingers naturally flexion, relaxing wrist joint, taking elbow as a fulcrum, active movement the forearm, driving wrist joint to swing rhythmically, so that the force generated is continuously acting on the Tui Na site through the thumb. At the same time, the subjects had a feeling of soreness and distension at the Hegu acupoint, and the manipulation frequency was about 120 times per minute (Fig. 1b).

**Fig. 2:**
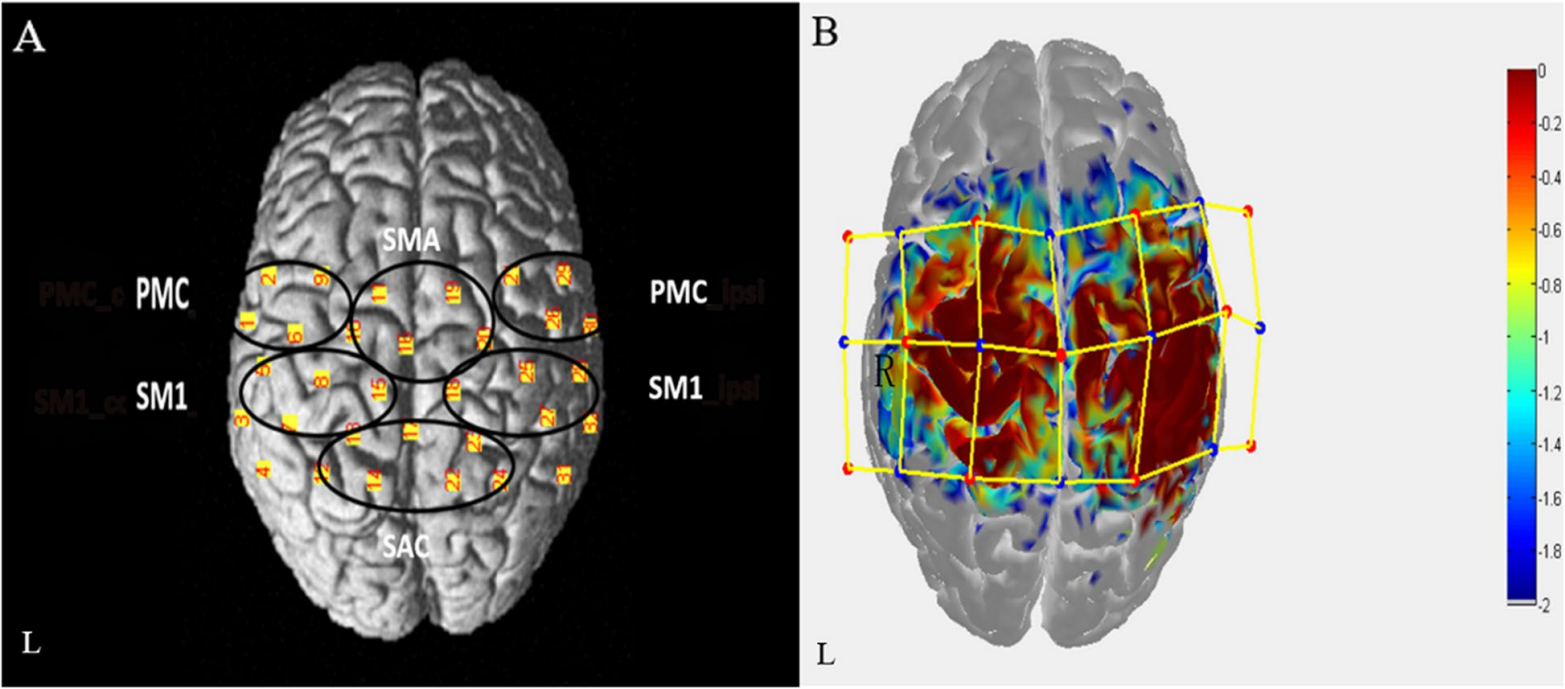
**A** The arrangement map of fNIRS channels in the cortex, which is divided into 6 ROIs. **B** Light sources (red dot, 11) and detectors (blue dot, 10) form 32 channels (yellow line). The sensitivity distribution map of photons in the cortex is simulated by Monte Carlo, and the redder the region is, the stronger the sensitivity is [19]

fNIRS equipment (NirScan-8000A, Danyang Huichuang, China) was used to record the hemodynamic data. A total of 21 NIRS optrodes (11 light sources and 10 detectors, with wavelengths of 730 and 850nm, and a sampling rate of 19Hz) formed 32 observation channels [19]. A three-dimensional digitizer (Patriot, America) was used to collect the 3D coordinate information of the optrodes and channels to ensure the accuracy of the observed brain area and to be consistent in multiple measurements. According to the Brodmann area (BA) and anatomical locations of the cortex, the observation channels are divided into six ROIs (regions of interest): ipsilesional premotor cortex, PMC_ipsi; contralesional premotor cortex, PMC_contra; ipsilesional primary sensorimotor cortex, SM1_ipsi; contralesional primary sensorimotor cortex, SM1_contra; supplementary motor area, SMA; Somatosensory Association Cortex, SAC[19] (Fig. 2).

### fNIRS data analysis

The fNIRS data was preprocessed and analyzed using NIRS – KIT [20] and NIRS-SPM v.3.2 [21, 22] based on MATLAB 2013b (The MathWorks Inc., Massachusetts). Considering that the oxygenated hemoglobin (Oxy-Hb) signal is widely reported in clinical research [23], with better sensitivity to task-related hemodynamic changes [24], and found excellent reliability for both task-related activity [25], we focused on Oxy-Hb signal in the present study.

For the correction of motion artifacts, the Temporal Derivative Distribution Repair (TDDR) algorithm was applied to the raw hemodynamic data [26]. Then, a discrete cosine transform (DCT) based high-pass filter (128 s) was applied to remove liner trends. Afterward, fNIRS data was smoothed with the canonical hemodynamic response function (HRF). The General Linear Model (GLM) was used to detect Tui Na-related brain activation from the preprocessed hemodynamic data. The design matrix consisting of a boxcar regressor of the massage task was convolved with a Gaussian HRF to obtain the predictors of the time series of neural activation. Beta-estimates represent the weight of the massage task to the variance of the hemodynamic signal of each channel. Cortical activation was calculated with a contrast vector of [1], and the significance level was set at *P <* 0.05. The number of activated channels in the contralateral and ipsilateral hemisphere was used to calculate the LI, LI = (contralateral − ipsilateral) / (contralateral + ipsilateral) [19].

### Statistical analysis

For demographic data, a group comparison of sex was carried out with a chi-square test, of age a two-tailed Wilcoxon test. For fNIRS data, comparisons between LI of stroke patients and controls were carried out with a two-tailed Wilcoxon test. Comparison between LI of affected arm Tui Na and less affected arm Tui Na among stroke patients was carried out with a one-tailed Wilcoxon test.

To reflect the differences more intuitively between groups and between conditions (Tui Na of affected / less affected arm), the preprocessed hemodynamic data of SM1 was z-score normalized and then averaged across groups and participants. The mean value of oxy-Hb changes during the 2-s period just before trial onset was subtracted from the trial average for baseline correction. Statistical significance was set at *p*<0.05.

## Results

### Demographic Information

As shown in Table 2, there were no significant differences in sex ratio and age between stroke patients and healthy controls.

**Table 2:** Demographic information

	**stroke patient (*N=*10)**	**healthy control (*N=*8)**	Significant
**Sex (male/female)**	8/2	5/3	0.41
**Age (years)**	58.00±10.31	49.25±6.82	0.06

*ST* Stroke patient, *HC* Healthy control

### Hegu acupoint Tui Na related fNIRS responses

**Fig. 3:**
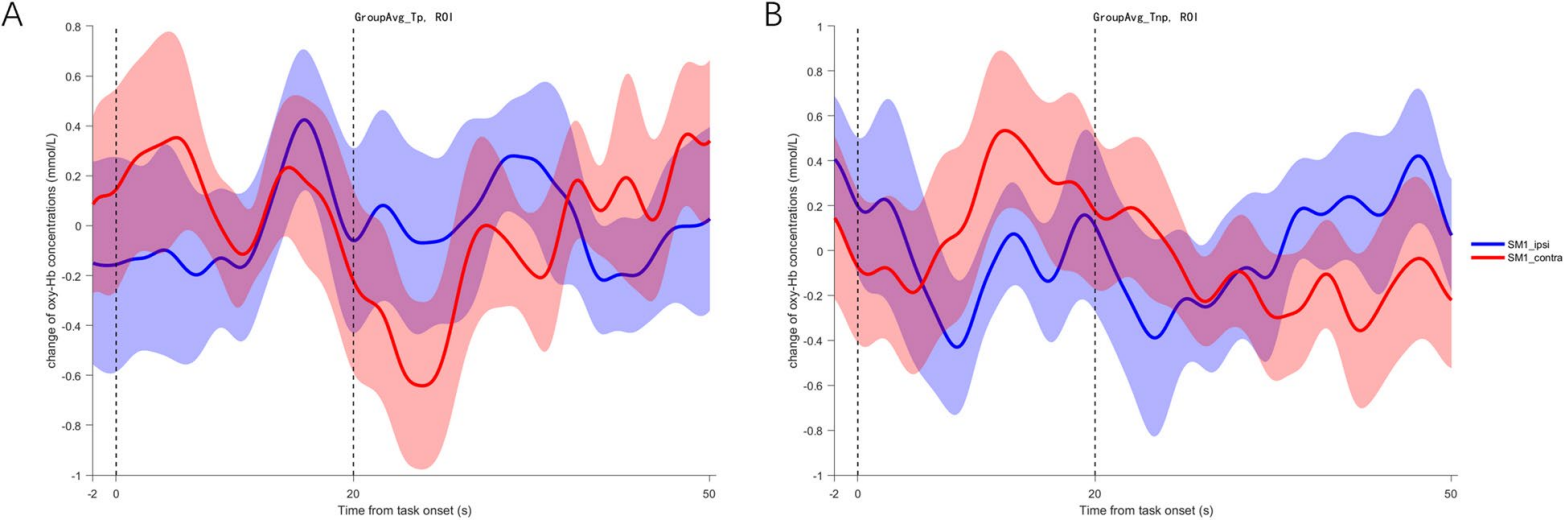
The averaged hemodynamic signal time courses in SM1 during Hegu acupoint Tui Na of affected (**A**) and less affected arm (**B**) in stroke patients. The blue line represents the ipsilesional hemisphere and the red for the contralesional hemisphere. The vertical dash lines indicate the onset and offset of the Tui Na task

**Fig. 4:**
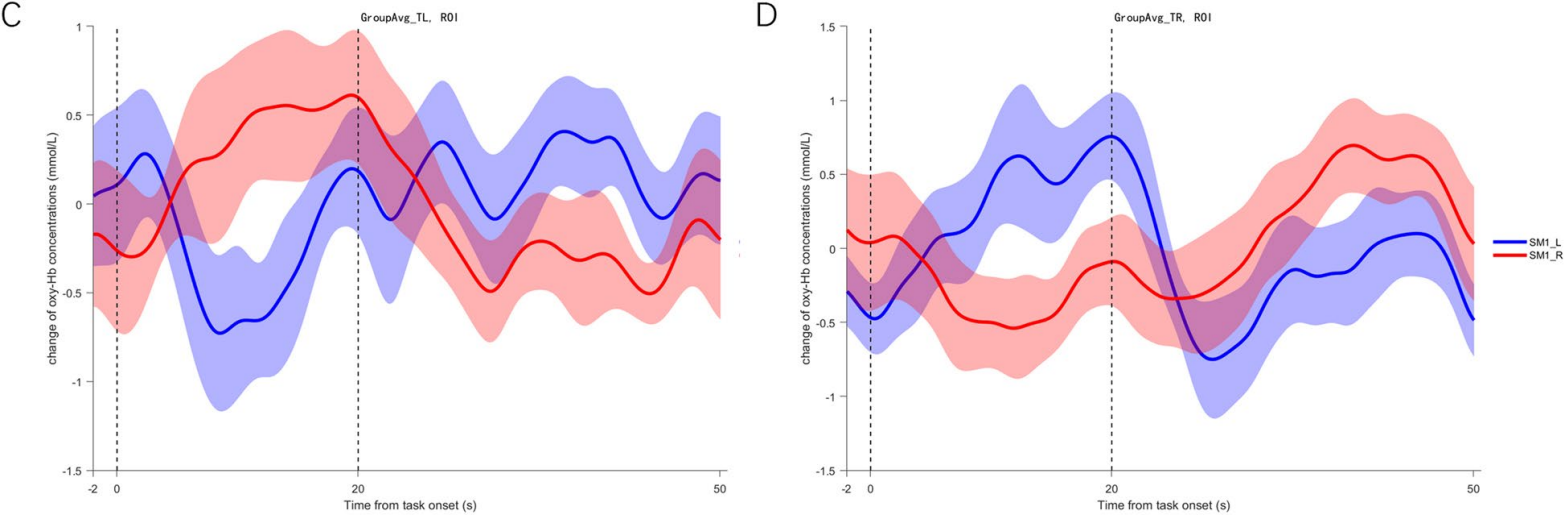
The averaged hemodynamic signal time courses in SM1 during Hegu acupoint Tui Na of the left (**C**) and right hand (**D**) in healthy controls. The blue line represents the left hemisphere and the red for the right hemisphere. The vertical dash lines indicate the onset and offset of the Tui Na task

Figure 3 showed a dominant hemodynamic increase in the contralesional SM1 during Hegu acupoint Tui Na of the less affected arm, while a similar pattern was not observed in the ipsilesional SM1 during Hegu acupoint Tui Na of the affected arm in stroke patients. In healthy controls, dominant hemodynamic increase in the contralateral SM1 was observed during Hegu acupoint Tui Na of both hands (Fig. 4).

### LI values

**Fig. 5:**
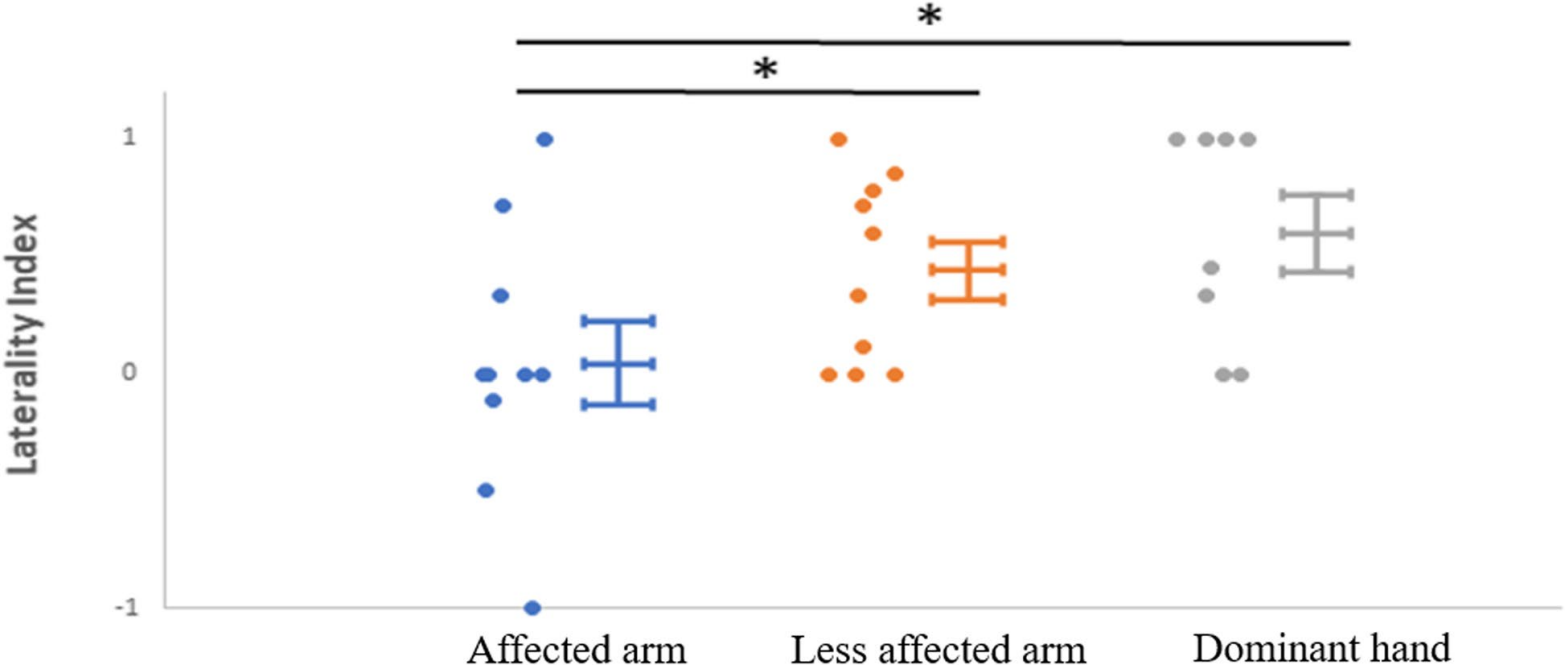
LI for the sensorimotor cortical regions during affected (left) and less affected (middle) arm Tui Na at Hegu acupoint, with mean values and standard deviation marked. On the left side, individual values are plotted. Values for healthy controls (dominant hand) are presented (right). ∗Significance level at *P <*0.05

Concerning the LI values in stroke patients, a significant difference was observed between LI values for the affected arm and the less affected arm (*P <* 0.05). As Fig. 5 shows, LI values for the affected arm at the Hegu acupoint varied from -1 to 1, indicating mixed patterns of sensorimotor cortical dysfunction and interhemispheric imbalance among stroke patients. Whereas, LI values for the less affected arm at Hegu acupoint in stroke patients were mostly above 0, suggesting the largely intact function of the contralesional sensorimotor cortex. Wilcoxon tests showed a significant difference between LI values for the affected arm in stroke patients and LI values for the dominant UL in healthy controls (*P <* 0.05), and no significant difference between LI values for the less affected arm in stroke patients and that in healthy controls (*P =*0.36).

## Discussion

The results of this study indicate that patients with UL motor dysfunction after stroke do have the injury of sensorimotor neural circuits, and the combination of Tui Na and fNIRS has the potential to reflect the dysfunction of sensorimotor neural circuits. The changes in neural activity in the sensorimotor cortex during Hegu acupoint Tui Na indicate that there is a certain correlation between acupoints in traditional Chinese medicine and neural circuits.

The pattern of brain activation of the affected limb after stroke was mainly bilateral in the early stage, and gradually transferred to the ipsilesional cortex with the progress of rehabilitation [27]. Therefore, the detection of LI may help the physician to make an auxiliary judgment on the prognosis of patients. Previous studies are mainly based on patients’ active hands-on tasks for related testing, but for patients who are unable to take the initiative tasks, there is a lack of detection, this study makes up for this deficiency.

The analysis of the effect of Tui Na on neural circuits is of great significance in the clinic. The neural circuits are the basis of the coordination of sensory, motor, and balance functions, and it is also the structure and functional unit of nerve remodeling and rehabilitation. The repair of these neural circuits after injury requires the stimulation of a variety of senses, and then reconstructing the neural network connection [28, 29]. Similar to acupuncture [30], Tui Na on the hemiplegic UL can stimulate the peripheral nerves, and promote the activation and integration of the cortical sensorimotor network. Similar to physical therapy [31], Tui Na can coordinate the balance of muscle tension between muscle groups, inhibit and control spasms, and then establish a normal movement pattern. As a non-invasive brain functional neuroimaging technology, fNIRS has the advantages of simple operation, low cost, strong anti-interference, and good compatibility. It can realize the rapid examination of brain function in healthy subjects and patients with various brain dysfunction diseases [32]. The commonly used non-invasive brain function detection techniques include electroencephalogram (EEG), functional magnetic resonance imaging (fMRI), and fNIRS. Each detection technique has its characteristics in spatial and temporal resolution, coverage, application requirements of the instrument, etc [33]. fMRI, which is widely used in clinical research, has high spatial resolution and can detect the whole brain, but it has a high cost and is more used for brain functional activity examination in a static state, so it is difficult to evaluate brain function in motor state or magnetoelectric therapy state [34]. Rehabilitation of nervous system diseases is a dynamic and gradual process and is closely related to brain function. The activation and reconstruction of neural circuits are crucial to realize functional rehabilitation [35]. Clinically, rehabilitation needs the process of "evaluation--rehabilitation--re-evaluation--continuous rehabilitation--curative effect follow-up", and needs to optimize the rehabilitation program continuously through feedback mechanism and clinical evaluation [36]. Therefore, it still needs real-time dynamic, portable, and easy-to-operate cortical function detection equipment. With its gradual popularization, fNIRS technology will show important application value and significance in the field of clinical research.

The limitations of this study are that the sample size is relatively small, Which may affect the accuracy of the study results. This study focuses on whether a single Tui Na massage at one point can cause changes in the sensorimotor cortex. And such a short and single session of Tui Na maybe not be enough to induce clinical improvement in dysfunction. In the future, we will use a session of a massage program and other physical therapy as a controlled study to operate at multiple time points, and to evaluate the UL dysfunction before and after therapy.

## Conclusion

The combination of Tui Na and fNIRS has the potential to reflect the functional status of sensorimotor neural circuits. The changes of neuroactivity in the sensorimotor cortex when Tui Na Hegu acupoint indicate that there is a certain correlation between acupoints in traditional Chinese medicine and neural circuits.

## Data Availability

Data of this study are available on request from the corresponding author.

## Abbreviations

fNIRS: Functional near-infrared spectroscopy
UL: Upper limb
LI: Lateralization index
Oxy-Hb: Oxygenated hemoglobin
SM1_ipsi: ipsilesional primary sensorimotor cortex
SM1_contra: contralesional primary sensorimotor cortex
HRF: Hemodynamic response function

## References

[1] Xu S, Yan Z, Pan Y, Yang Q, Liu Z, Gao J, Yang Y, Wu Y, Zhang Y, Wang J, Zhuang R, Li C, Zhang Y, Jia J (2021). Associations between Upper Extremity Motor Function and Aphasia after Stroke: A Multicenter Cross-Sectional Study. Behav Neurol.

[2] Tang Y, Wang L, He J, Xu Y, Huang S, Fang Y (2021). Optimal Method of Electrical Stimulation for the Treatment of Upper Limb Dysfunction After Stroke: A Systematic Review and Bayesian Network Meta-Analysis of Randomized Controlled Trials. Neuropsychiatr Dis Treat.

[3] Cabanas-Valdés R, Calvo-Sanz J, Serra-Llobet P, Alcoba-Kait J, González-Rueda V, Rodríguez-Rubio PR (2021). The Effectiveness of Massage Therapy for Improving Sequelae in Post-Stroke Survivors. A Systematic Review and Meta-Analysis. Int J Environ Res Public Health.

[4] Kovich F (2019). A New Definition of an Acupuncture Meridian. J Acupunct Meridian Stud.

[5] Chen X, Sun X, Liu N, Wang M, Tang W, Jiang Y, Bryan M, Cai Y (2021). Analysis of Human Acupoint Biological Information and Neural Electric Activity Based on Ultrasonographic Image. World Neurosurg.

[6] Wang J, Cui JJ, Ha LJ, She C, Xu DS, Jing XH, Yu XC, Bai WZ (2019). Review on application of neural tracing technique to experimental research of acupuncture. Zhen Ci Yan Jiu..

[7] Ferguson MA, Schaper FLWVJ, Cohen A, Siddiqi S, Merrill SM, Nielsen JA, Grafman J, Urgesi C, Fabbro F, Fox MD (2022). A Neural Circuit for Spirituality and Religiosity Derived From Patients With Brain Lesions. Biol Psychiatry.

[8] Kazim SF, Bowers CA, Cole CD, Varela S, Karimov Z, Martinez E, Ogulnick JV, Schmidt MH (2021). Corticospinal Motor Circuit Plasticity After Spinal Cord Injury: Harnessing Neuroplasticity to Improve Functional Outcomes. Mol Neurobiol.

[9] Wang M, Liu S, Peng Z, Zhu Y, Feng X, Gu Y, Sun J, Tang Q, Chen H, Huang X, Hu J, Chen W, Xiang J, Wan C, Fan G, Lu J, Xia W, Chen L, Wang L, Lu X, Li J (2019). Effect of Tui Na on upper limb spasticity after stroke: a randomized clinical trial. Ann Clin Transl Neurol.

[10] Yang YJ, Zhang J, Hou Y, Jiang BY, Pan HF, Wang J, Zhong DY, Guo HY, Zhu Y, Cheng J (2017). Effectiveness and safety of Chinese massage therapy (Tui Na) on post-stroke spasticity: a prospective multicenter randomized controlled trial [J]. Clin Rehabil.

[11] Arun KM, Smitha KA, Sylaja PN, Kesavadas C (2020). Identifying Resting-State Functional Connectivity Changes in the Motor Cortex Using fNIRS During Recovery from Stroke. Brain Topogr.

[12] Boas DA, Elwell CE, Ferrari M, Taga G (2014). Twenty years of functional near-infrared spectroscopy: introduction for the special issue. NeuroImage.

[13] Nguyen HD, Hong KS (2016). Bundled-optode implementation for 3D imaging in functional near-infrared spectroscopy [J]. Biomed Opt Express.

[14] Zafar A, Hong KS (2017). Detection and classification of three-class initial dips from prefrontal cortex. Biomed Opt Express.

[15] Ghafoor U, Lee JH, Hong KS, Park SS, Kim J, Yoo HR (2019). Effects of Acupuncture Therapy on MCI Patients Using Functional Near-Infrared Spectroscopy. Front Aging Neurosci.

[16] Curtin A, Ayaz H, Tang Y, Sun J, Wang J, Tong S (2019). Enhancing neural efficiency of cognitive processing speed via training and neurostimulation: An fNIRS and TMS study. Neuroimage.

[17] Li C, Su M, Xu J, Jin H, Sun L (2020). A Between-Subject fNIRS-BCI Study on Detecting Self-Regulated Intention During Walking. IEEE Trans Neural Syst Rehabil Eng.

[18] Almulla L, Al-Naib I, Althobaiti M (2020). Hemodynamic responses during standing and sitting activities: a study toward fNIRS-BCI. Biomed Phys Eng Express.

[19] Chen Y, Zhu G, Mao M, Zheng Y, Huang H, Liu L, Chen S, Cao L, Xu D (2022). Study protocol of a randomized controlled trial for the synergizing effects of rTMS and Tui Na on upper limb motor function and cortical activity in ischemic stroke. Front Neurol..

[20] Hou X, Zhang Z, Zhao C, Duan L, Gong Y, Li Z, Zhu C (2021). NIRS-KIT: a MATLAB toolbox for both resting-state and task fNIRS data analysis. Neurophotonics.

[21] Ye JC, Tak S, Jang KE, Jung J, Jang J (2009). NIRS-SPM: Statistical parametric mapping for near-infrared spectroscopy. NeuroImag.

[22] Tsuzuki D, Dan I (2014). Spatial registration for functional near-infrared spectroscopy: from channel position on the scalp to cortical location in individual and group analyses. Neuroimage.

[23] Ozawa S (2021). Application of Near-Infrared Spectroscopy for Evidence-Based Psychotherapy. Front Psychol.

[24] Yeung MK, Lin J (2021). Probing depression, schizophrenia, and other psychiatric disorders using fNIRS and the verbal fluency test: A systematic review and meta-analysis. J Psychiatr Res.

[25] Huang Y, Mao M, Zhang Z, Zhou H, Zhao Y, Duan L (2017). Test-retest reliability of the prefrontal response to affective pictures based on functional near-infrared spectroscopy. J Biomed Opt.

[26] Fishburn FA, Ludlum RS, V aidya CJ, Medvedev AV (2019). Temporal Derivative Distribution Repair (TDDR): A motion correction method for fNIRS. Neuroimage.

[27] Delorme M, Vergotte G, Perrey S, Froger J, Laffont I (2019). Time course of sensorimotor cortex reorganization during upper extremity task accompanying motor recovery early after stroke: An fNIRS study. Restor Neurol Neurosci..

[28] Szczesna A, Blaszczyszyn M, KAW ALA-STERNIUK A (2020). Convolutional neural network in UL functional motion analysis after stroke. PeerJ.

[29] Huang J (2020). Dynamic activity of human brain task-specific networks. Sci Rep.

[30] Schaechter JD, Connell BD, Stason WB, Kaptchuk TJ, Krebs DE, Macklin EA, Schnyer RN, Stein J, Scarborough DM, Parker SW, McGibbon CA, Wayne PM (2007). Correlated change in upper limb function and motor cortex activation after verum and sham acupuncture in patients with chronic stroke. J Altern Complement Med..

[31] Gopaul U, van Vliet P, Callister R, Nilsson M, Carey L (2019). Conbined Physical and somatosensory training after stroke: Development and description of a novel intervention to improve upper limb function. Physiother Res Int..

[32] Huo C, Xu G, Li Z, Lv Z, Liu Q, Li W, Ma H, Wang D, Fan Y (2019). Limb linkage rehabilitation training-related changes in cortical activation and effective connectivity after stroke: A functional near-infrared spectroscopy study [J]. Sci Rep.

[33] Anwar AR, Muthalib M, Perrey S, Galka A, Granert O, Wolff S, Heute U, Deuschl G, Raethjen J (2016). Muthuraman M Effective Connectivity of Cortical Sensorimotor Networks During Finger Movement Tasks: A Simultaneous fNIRS, fMRI, EEG Study [J]. Brain Topogr.

[34] Yousaf T, Dervenoulas G, Politis M (2018). Advances in MRI Methodology. Int Rev Neurobiol.

[35] W arraich Z, Kleim JA (2010). Neural plasticity: the biological substrate for neurorehabilitation. PM R.

[36] Marchiafava M, Bedetti C, Buratta L, Ilicini S, Cicuttin S, Piccirilli M, d'Alessandro P, Baglioni A, Menna M, Gubbiotti M, Lanfaloni GA, Rossi MG, Elisei S (2019). Evaluation of the Quality of Rehabilitation Treatment in Neurodevelopmental Disorder. Psychiatria Danubina..

